# Chemical species recognition in an adaptive radiation of Hawaiian *Tetragnatha* spiders (Araneae: Tetragnathidae)

**DOI:** 10.1098/rspb.2023.2340

**Published:** 2024-04-10

**Authors:** Seira A. Adams, Anjali Gurajapu, Albert Qiang, Moritz Gerbaulet, Stefan Schulz, Neil D. Tsutsui, Santiago R. Ramirez, Rosemary G. Gillespie

**Affiliations:** ^1^ Department of Environmental Science, Policy, and Management, University of California, 130 Mulford Hall, #3114, Berkeley, CA 94720, USA; ^2^ Center for Population Biology, University of California, 2320 Storer Hall, Davis, CA 95616, USA; ^3^ Department of Evolution and Ecology, University of California, 2320 Storer Hall, Davis, CA 95616, USA; ^4^ Institute of Organic Chemistry, Technische Universität Braunschweig, Hagenring 30, Braunschweig 38106, Germany

**Keywords:** speciation, reproductive isolation, pheromones, lipids, methyl ethers, silk

## Abstract

Studies of adaptive radiations have played a central role in our understanding of reproductive isolation. Yet the focus has been on human-biased visual and auditory signals, leaving gaps in our knowledge of other modalities. To date, studies on chemical signals in adaptive radiations have focused on systems with multimodal signalling, making it difficult to isolate the role chemicals play in reproductive isolation. In this study we examine the use of chemical signals in the species recognition and adaptive radiation of Hawaiian *Tetragnatha* spiders by focusing on entire communities of co-occurring species, and conducting behavioural assays in conjunction with chemical analysis of their silks using gas chromatography-mass spectrometry. Male spiders significantly preferred the silk extracts of conspecific mates over those of sympatric heterospecifics. The compounds found in the silk extracts, long chain alkyl methyl ethers, were remarkably species-specific in the combination and quantity. The differences in the profile were greatest between co-occurring species and between closely related sibling species. Lastly, there were significant differences in the chemical profile between two populations of a particular species. These findings provide key insights into the role chemical signals play in the attainment and maintenance of reproductive barriers between closely related co-occurring species.

## 1. Introduction

Studies of adaptive radiations have been central in developing our understanding of the processes involved in speciation and diversification. The explosive nature of adaptive radiations that result in exceptionally high levels of diversity, often with multiple, closely related species co-occurring with one another, has provided fruitful grounds for biologists to study these evolutionary processes [1,2]. Specifically, these systems have played a key role in understanding how divergence between close relatives is attained and maintained, highlighting the complex interplay between ecological and reproductive isolation. While some studies have highlighted ecological shifts as drivers of divergence [2–5], many others have identified sexual recognition cues as playing a central role [2,6–11]. While the conflicting results may reflect fundamental differences between lineages, it may also be due to some signals (especially visual and auditory) being readily amenable to measurement while those that fall outside human sensory modalities are much more difficult to quantify. Here, we examine the possibility that chemical differences might serve as hidden cues for reproductive isolation in adaptive radiations.

While chemical cues are well known to play an essential role in mate and resource choice in many taxa [12–14], their role as an isolating mechanism in adaptive radiation is largely unknown. Pioneering work on moths and *Drosophila* have shown that rapid evolution of chemical signals is important in the early stages of reproductive isolation [15,16]. Chemical studies on the adaptive radiation of *Heliconius* butterflies [17–20], Hawaiian *Drosophila* [21,22], Hawaiian *Laupala* crickets [23,24] and Cameroonian cichlid fish [25] show a similar result. Nevertheless, it has been difficult to untangle the relative significance of chemical communication in reproductive isolation in relation to the other seemingly more dominant visual and auditory forms of communication integral to these systems, and the extent to which chemicals may provide sufficient species specificity to isolate co-occurring species. The current study examines an adaptive radiation of spiders where multiple closely related species co-occur despite exhibiting little to no evidence for the use of visual or auditory communication [26,27], with hybridization between co-occurring species being uncommon [28]. This work takes a novel approach in examining the role of chemical signals in reproductive isolation by focusing on entire communities of co-occurring species within a rapidly diversifying lineage where the relative contribution of chemical signals can be studied in isolation from other communication modalities.

The Hawaiian Islands are well known for many examples of adaptive radiation. Among spiders, the larger radiations are seen in nocturnal taxa [29]. The spider genus *Tetragnatha*, in particular, includes over 50 different species distributed across the archipelago. While generally known for their drab coloration, elongate body, and a propensity to build horizontal orb webs over water [30], representatives in the Hawaiian Islands exhibit a wide array of morphologies and ecologies. The Hawaiian species are divided into two major clades: the ‘web-building’ clade that builds orb webs, and the ‘spiny leg’ clade that has abandoned web-building and lives a cursorial lifestyle [26,27,31–33]. Spiders from both clades produce the characteristic spider dragline silk, a silk type used in mobility, but differ in the use of an orb web for prey capture. Up to eight representatives from these two clades co-occur in the native wet and mesic forests on each of the current high islands [26,27]. However, like their mainland counterparts, they have low visual ability, are exclusively nocturnal, and show little evidence of vibratory communication, even among the web-builders, which generally have flimsy webs [30] with the strands often water-drenched, making them seemingly unfit to transmit vibratory cues [27]. Moreover, the spiders lack stridulatory structures [26,27,30,34], which are commonly found in non-web building spiders and used for vibratory communication [35–37]. Yet, despite the lack of obvious signaling modalities, there is little evidence of hybridization [28] and populations of sister taxa are almost invariably highly structured [28,38]. The lack of evidence for either visual or vibrational cues in courtship led to the assumption that the diversification was driven by ecological processes, although the means through which sister taxa achieved reproductive isolation is unclear [31,32]. These observations raised the question of whether chemicals signals might provide a mechanism for communication, species recognition and reproductive isolation among co-occurring species.

Evidence for the use of chemical signals in spiders is well documented in intraspecific mate choice experiments, showing the ubiquitous use of chemicals across the spider phylogeny [37,39,40]. Furthermore, in interspecific interactions, studies conducted on *Linyphia* and distantly related (European and North American) *Tetragnatha* spiders have demonstrated that long chain alkyl methyl ether lipids found on the silk of female spiders play a role in distinguishing conspecific from heterospecific mates [40–42]. From this we can hypothesize that these methyl ethers may play a role in species recognition between closely related co-occurring species of Hawaiian *Tetragnatha*. Here we investigate the role of methyl ethers in the species recognition of co-occurring Hawaiian *Tetragnatha* spiders and determine the extent of the species-specific signals, how the compounds in the signals have evolved structurally, and whether the signals differ between populations of a given species. Specifically, we examine three hypotheses: (1) methyl ethers derived from the silk allow species to behaviorally distinguish conspecifics from co-occurring heterospecifics; (2) co-occurring species have methyl ethers that differ either in structural identity or in abundance; and (3) if chemicals play a role in species recognition, then they will differ most between close relatives in sympatry compared to those in allopatry. We find that male Hawaiian *Tetragnatha* spiders from both the web-building and spiny leg clades use chemicals found on the silk of females to distinguish conspecifics from co-occurring heterospecifics, and that these chemicals are species-specific in the structural identity and not simply the amount of the compounds. In comparison, species in allopatry showed considerable overlap in their chemical signatures. Our results highlight the potential key role of chemicals in reproductive isolation between closely related co-occurring species in an adaptive radiation where other communication modalities appear nonessential.

## 2. Results

### (a) Chemicals derived from silk allowed species to discriminate conspecifics from sympatric heterospecifics

**Figure 1.**
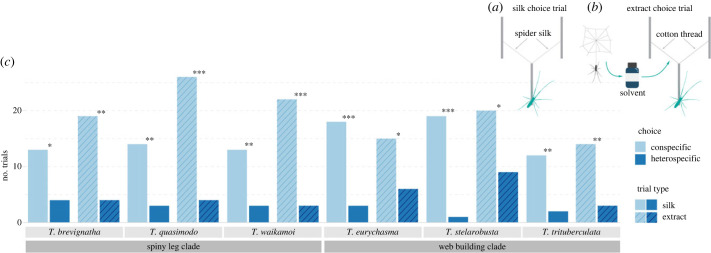
Results for male silk and silk extract choice trials. (*a*) Silk choice trial and (*b*) silk extract choice trial set-ups (not drawn to scale). Males were released at the far end of the center stick. (*c*) Results of male choice when given a choice between conspecific versus heterospecific female silk in the silk choice trials (silk—solid bars), and the chemical extract of conspecific versus heterospecific female silk in the silk extract choice trials (extract—hatched bars). All trials were significant with the degree of significance indicated by the star above each plot (* = *p* ≤ 0.05, ** = *p* ≤ 0.01, *** = *p* ≤ 0.001). Results for silk choice trials were: *T. brevignatha*: *χ*^2^ = 4.765, df = 1, *p* = 0.02905, 13 : 4; *T. quasimodo*: *χ*^2^ = 7.118, df = 1, *p* = 0.008, 14 : 3; *T. waikamoi*: *χ*^2^ = 6.25, df = 1, *p* = 0.012, 13 : 3; *T. eurychasma*: *χ*^2^ = 10.714, df = 1, *p* = 0.001, 18 : 3; *T. stelarobusta*: *χ*^2^ = 16.2, df = 1, *p* = < 0.001, 19 : 1; *T. trituberculata*: *χ*^2^ = 7.143, df = 1, *p* = 0.008, 12 : 2. Results for silk extract choice trials were: *T. brevignatha*: *χ*^2^ = 9.783, df = 1, *p* = 0.002, 19 : 4; *T. quasimodo*: *χ*^2^ = 16.133, df = 1, *p* = < 0.001, 26 : 4; *T. waikamoi*: *χ*^2^ = 14.44, df = 1, *p* = < 0.001, 22 : 3; *T. eurychasma*: *χ*^2^ = 3.857, df = 1, *p* = 0.049, 15 : 6; *T. stelarobusta*: *χ*^2^ = 4.172, df = 1, *p* = 0.041, 20 : 9; *T. trituberculata*: *χ*^2^ = 7.118, df = 1, *p* = 0.008, 14 : 3.

Silk choice trials and silk extract choice trials were conducted to determine whether males can discriminate the silk or silk extract of conspecific females from that of sympatric heterospecific females. For all six species of Hawaiian *Tetragnatha*, males selected the silk of the conspecific female at a significantly higher frequency than that of the sympatric heterospecific female (figure 1). Similar results were found in the silk extract choice trials, where males selected the extract of the conspecific female at a significantly higher frequency than that of the sympatric heterospecific female (figure 1). The same pattern was observed in silk choice trials and silk extract choice trials between sympatric species from the same clade (web-building or spiny leg) and between sympatric species from different clades (figure 1; electronic supplementary material, S1).

### (b) Sympatric species had methyl ethers that were unique and species-specific

The compounds found in the silk extracts were analysed along the dimensions of chemical identity, proportion, and structure (for example, the chain length and methyl branch position), as these components have been shown to be crucially important in mate recognition and reproductive isolation [12,13].

**Figure 2.**
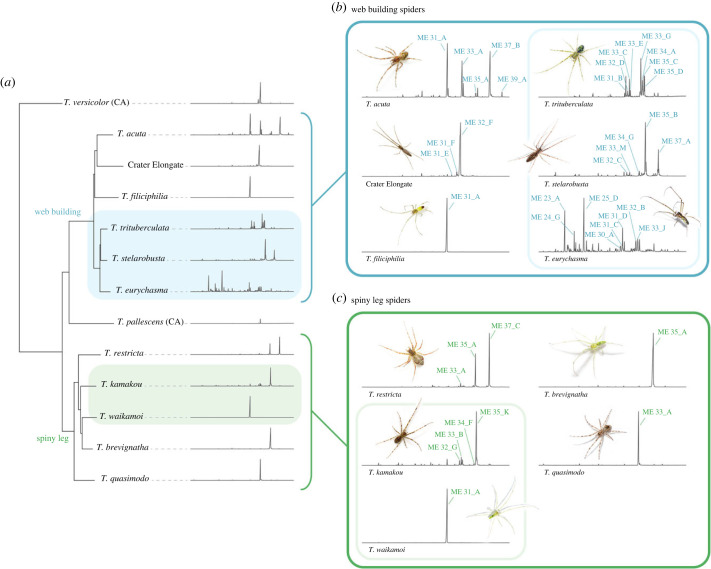
Representative chemical profiles of each species. (*a*) The GC-MS chromatograms of representative dragline extracts from females of each species positioned next to a reduced phylogenetic tree. The web building clade is indicated in the dark blue while the spiny leg clade is indicated in the dark green colour. The light blue and light green blocks highlight the closely related species that co-occur in both the web building and spiny leg clades respectively. (*b*) A closer look at the chromatograms of Hawaiian *Tetragnatha* spiders from the web-building clade arranged in the same order as the phylogenetic tree (from top left down to top right down). The most abundant methyl ethers are labelled with their specific shorthand code and the light blue box surrounds the closely related sympatric web building species that can be found in Upper Waikamoi. (*c*) The same as (*b*) but for spiders from the spiny leg clade. The most abundant methyl ethers are labelled with their specific shorthand code and the light green box surrounds the closely related sympatric spiny leg species that can be found in Upper Waikamoi.

A total of 124 unique methyl ethers were detected in the dragline silk extracts of the 11 Hawaiian *Tetragnatha* species (six web-building and five spiny leg species), and the two Californian web-building *Tetragnatha* species that were analysed (electronic supplementary material, S2). The Hawaiian web-building clade overall had significantly more methyl ether compounds in their profile than both the Hawaiian spiny leg and Californian species (average number of methyl ethers: Hawaiian web-building = 4.84 ± 3.66, Hawaiian spiny leg = 2.15 ± 1.64, California web-building = 3.24 ± 2.89; ANOVA: *F*_2,239_ = 24.67, *p* = < 0.001) (electronic supplementary material, S3). For each of the 13 species, the compounds with the highest relative abundance and/or most consistent presence (detected in over 50% of individuals) were recorded, resulting in a total of 32 methyl ethers across all species which underwent further structural analysis. Of the remaining 92 unique methyl ethers, 61 were found in only one or two individuals and the rest were found in low relative abundance across multiple individuals (figure 2; electronic supplementary material, S2 and S4).

The structures of the 32 methyl ethers were elucidated by combined analysis of their mass spectra, gas chromatographic retention indices and microderivatization of extracts by transformation into cyanides and methyl esters (electronic supplementary material, S5 and S6) [42–44]. The latter was necessary because the location of methyl groups along the alkyl chain cannot be determined by analysis of the mass spectra of the native methyl ethers. Methyl ethers ranged in size from 22 to 39 total carbons, while the number of methyl branches ranged from as few as one to as many as five. Hierarchical clustering of the methyl ethers based on maximum common substructure suggested an optimal number of six methyl ether groups (electronic supplementary material, S7–S10). When the methoxy group was accounted for in the analysis, groups were defined mostly by the carbon backbone length (electronic supplementary material, S9 and S10). When the methoxy group was excluded from the analysis, the shared methyl branch positions were a stronger factor in the clustering, and the resulting clusters were more species-specific (electronic supplementary material, S7 and S8). The ancestral state reconstruction of the ω-methyl branch pattern (a way to count the methyl branch position from the opposite end of the methoxy group) revealed that even-numbered ω-branch positions were the most common and ancestral form found in both Hawaiian clades and the Californian outgroup, while odd-numbered ω-branch positions were only found in the Hawaiian web-building clade (electronic supplementary material, S11). Across all the species, the most common branch positions were ω-10, ω-16 and ω-22, with ω-16 found in all species except for *T. stelarobusta* (figure 5*a*).

**Figure 3.**
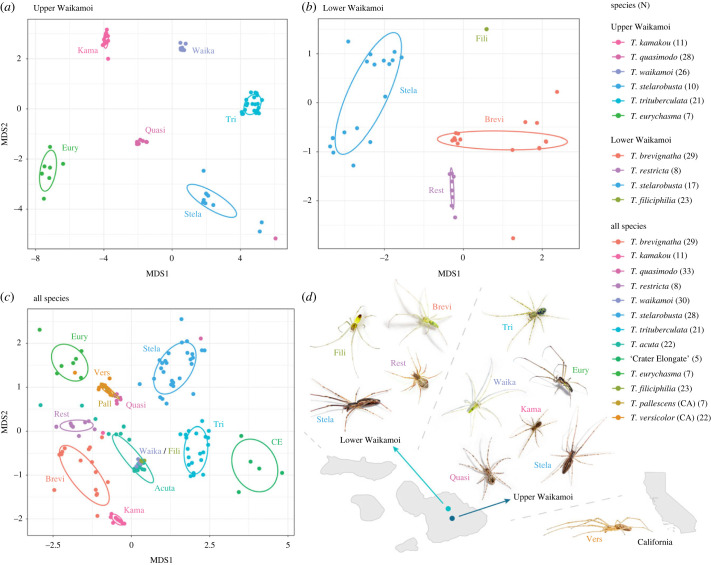
Chemical profile of sympatric individuals and all individuals sampled. NMDS plot of the chemical profiles of the Hawaiian *Tetragnatha* species found in sympatry at (*a*) Upper Waikamoi (stress = 0.024) and at (*b*) Lower Waikamoi (stress = 0.031), as well as a NMDS plot of (*c*) the chemical profiles of all spider species: 11 Hawaiian *Tetragnatha* species and 2 Californian species (stress = 0.049). Each dot represents an individual and the number of individuals for each species is listed next to the species name in the key. (*d*) Location of Upper Waikamoi and Lower Waikamoi on Maui and images of the representative spiders from those two sites and California.

The NMDS results (figure 3; electronic supplementary material, S12) showed that the chemical profiles of individual spiders (*n* = 246) clustered strongly based on species, suggesting that the identities and amounts of methyl ethers found in the profiles were highly species-specific. There was very little overlap in the chemical profiles between different species, with the few exceptions being between species that do not co-occur. *Tetragnatha quasimodo* and *T. versicolor* showed significant overlap due to 1-methoxy-8,14,20-trimethylnonacosane (ME 33_A) being a major contributor to both species' profiles, while *T. acuta*, *T. waikamoi and T. filiciphilia* had overlapping chemical profiles with 1-methoxy-6,12,18-trimethylheptacosane (ME 31_A) shared across these species. ME 31_A was the only major contributor to the chemical profiles of *T. waikamoi* and *T. filiciphilia*, leading to a near perfect overlap on the NMDS plot (figure 3c). PERMANOVA and PERMDISP analysis further supported a strong species effect on chemical profile (ADONIS: *F*_12,227_ = 57.55, *R*^2^ = 0.753, *p* = 0.001; permutest: *F*_12,227_ = 14.359, *p* = 0.001) (electronic supplementary material, S13 and S14). Between male and female specimens of the same species, PERMANOVA analysis showed that chemical profiles differed significantly between the two sexes in *T. acuta*, *T. brevignatha*, *T. stelarobusta* and *T. trituberculata* (electronic supplementary material, S15), with the differences being the most remarkable in *T. brevignatha*. In *T. brevignatha* 1-methoxy-8,16,22-trimethylhentriacontane (ME 35_A) was the major compound found in female samples while 1-methoxy-6,14,20-trimethylnonacosane (ME 33_B) was the major compound in male samples (S15).

**Figure 4.**
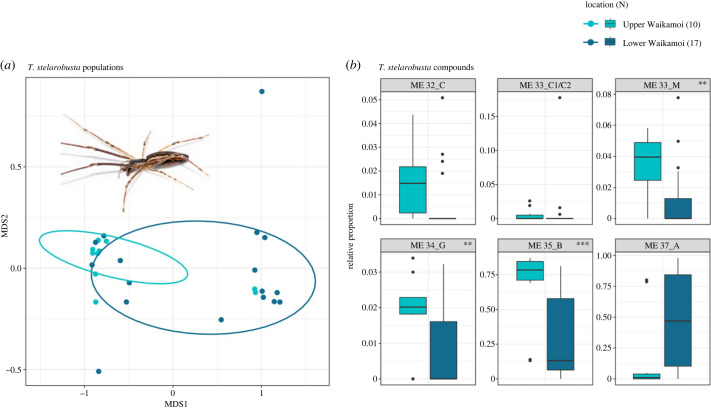
Chemical difference between two populations. (*a*) NMDS plot of the chemical profiles of the two populations of *T. stelarobusta* collected from Upper Waikamoi and Lower Waikamoi. (*b*) Box and whisker plot of the relative proportion of specific methyl ethers found in the individual profiles of *T. stelarobusta* from Upper and Lower Waikamoi. The degree of significance is indicated by the star above each plot (* = *p* ≤ 0.05, ** = *p* ≤ 0.01, *** = *p* ≤ 0.001). The results for each methyl ether compound were: ME 32_C: *F*_1,25_ = 2.223, *p* = 0.148; ME 33_C1/C2: *F*_1,25_ = 0.224, *p* = 0.64; ME 33_M: *F*_1,25_ = 6.301, *p* = 0.019; ME 34_G: *F*_1,25_ = 6.63, *p* = 0.016; ME 35_B: *F*_1,25_ = 8.188, *p* = 0.008; ME 37_A: *F*_1,25_ = 4.03, *p* = 0.056.

*Tetragnatha stelarobusta*, the only species sampled at both the Upper Waikamoi and Lower Waikamoi sites, exhibited population-specific differences in chemical profiles with some overlap, as revealed in the PERMANOVA and PERMDISP analysis (ADONIS: *F*_1,25_ = 4.848, *R*^2^ = 0.162, *p* = 0.027; permutest: *F*_1,25_ = 4.033, *p* = 0.051) (figure 4). Furthermore, *t*-tests revealed that the relative abundances of the methyl ethers found in the chemical profiles were significantly different in the two populations, with higher abundances of 1-methoxy-4,10,16,20,24-pentamethylheptacosane (ME 33_M), 1-methoxy-4,10,16,22,26-pentamethyloctacosane (ME 34_G) and 1-methoxy-4,10,16,22,26-pentamethylnonacosane (ME 35_B) in Upper Waikamoi (figure 4).

### (c) Sympatric species exhibited greater chemical disparity than allopatric species

The disparity through time (DTT) analysis revealed a high level of within-subclade disparity in chemical profiles throughout the phylogeny, showing a pattern of chemical profile evolution that diverges from Brownian motion (electronic supplementary material, S16). The three-dimensional traitgram provides a visualization of this divergence, with closely related species occupying drastically different regions in the NMDS space (electronic supplementary material, S17). When comparing the chemical distances among sympatric species pairs and allopatric species pairs, the overall distribution was significantly different, with sympatric species pairs being more tightly distributed at higher chemical distance values relative to allopatric species pairs (variance: allopatric = 0.053; sympatric = 0.007; Fligner–Killeen: chi-square = 11.837, d.f. = 1, *p* = < 0.001) (S18–S19).

**Figure 5.**
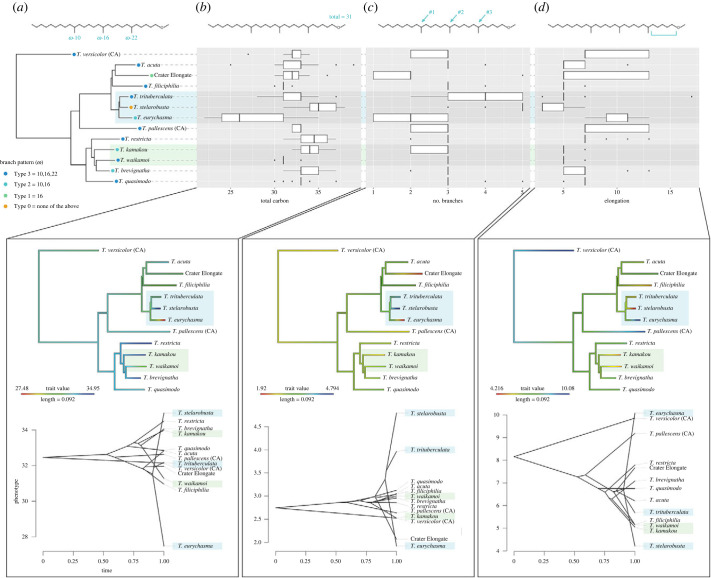
Structural characteristics of the most common methyl ethers found in each species. (*a*) The prominent omega (ω) methyl branch patterns of the methyl ethers found in each species is mapped onto a phylogenetic tree where ‘Type 3’ indicates *Tetragnatha* species that have methyl ethers with a branch on position ω-10, ω-16 and ω-22, ‘Type 2’ with a branch on position ω-10 and ω-16, ‘Type 1’ with a branch on position ω-16, and lastly ‘Type 0’ species that do not have any branches on position ω-10, ω-16 nor ω-22. (*b-d*) A box and whisker plot, character mapping conducted on the phylogeny, and traitgram projections representing (*b*) the total number of carbons, including the methoxy group, in each compound, (*c*) the number of methyl branches on each compound, and (*d*) the elongation of each compound. The closely related sympatric species of web-builders are highlighted in light blue, while spiny legs are highlighted in light green.

The structures of the methyl ethers were significantly different among the most closely related sympatric species in both the web-building clade and spiny leg clade (figure 5). Of the closely related sympatric web-building species, *T. eurychasma*, *T. stelarobusta* and *T. trituberculata*, significant structural divergence was observed in the methyl ethers between *T. eurychasma* and *T. stelarobusta*, with *T. eurychasma* having methyl ethers that were smallest in size, contained the fewest number of branches, and had the longest elongation. By contrast, *T. stelarobusta* had methyl ethers that were largest in size, contained the most branches, and had the shortest elongation (figure 5; electronic supplementary material, S20 and S21). Even when examining all 13 species, the divergence between *T. eurychasma* and *T. stelarobusta* continued to be pronounced, with each found at opposite ends of the entire trait spectrum (figure 5). Occupying a middle ground between these two extremes, *T. trituberculata* was intermediate in all three dimensions while still being significantly different from *T. eurychasma* and *T. stelarobusta* (figure 5; electronic supplementary material, S20 and S21). Furthermore, while many of the ω-branch patterns were shared across multiple species, the three sympatric web-building species each exhibited a distinct pattern from one another (figure 5a). Maximum common substructure analysis further supported the divergence between *T. eurychasma* and *T. stelarobusta*, with each containing methyl ethers that separated into significantly different groupings (electronic supplementary material, S7 and S8). Although less pronounced, significant structural divergence was also found between the closely related sympatric spiny leg species, *T. kamakou* and *T. waikamoi*, with *T. kamakou* having methyl ethers that were larger in size and contained fewer number of branches (figure 5; electronic supplementary material, S20 and S21). The ω-branch patterns of the two species were also distinct, and again, the maximum common substructure analysis revealed their methyl ethers separating into significantly different groupings (electronic supplementary material, S7–S10).

## 3. Discussion

Our study shows that males of Hawaiian *Tetragnatha* spiders use chemical signals to discriminate between conspecific and co-occurring heterospecific females. Behavioural analysis showed that males preferred the silk extract of conspecific females over those of sympatric heterospecific females (figure 1). In addition, where multiple species co-occur, these chemical signals are highly species-specific showing no overlap between any of the species (figure 3). Moreover, there were significant differences in the fundamental structure of these compounds, with higher differentiation between closely related sympatric species as opposed to those occurring in allopatry (figure 5).

Chemical signals found on the silk of *Tetragnatha* spiders showed a clear role in species recognition. Behavioural results showed that species from both the web-building clade and the spiny leg clade were able to distinguish conspecific from sympatric heterospecific mates, indicating the widespread nature of the use of chemicals in species recognition in Hawaiian *Tetragnatha* spiders, whether or not they build capture webs. Even when males were tested with sympatric heterospecific females from a different clade to their own, the results were the same as when males were tested with sympatric heterospecific females from their own clade. This suggests that chemical signals allow individuals to differentiate conspecific mates from all other *Tetragnatha* species found within a given community which is often composed of a mix of both web-building and spiny leg species. A key finding of the current study was that, overall, there was very little overlap in the methyl ethers between species and most methyl ethers were unique to a given species. Studies in other adaptive radiations, including *Heliconius* butterflies [17–20], Hawaiian *Drosophila* [21], and Hawaiian *Laupala* crickets [23,24] have also shown that the chemical signals between coexisting species are divergent. However, many compounds can be found shared across multiple species, as seen in the *Heliconius* butterflies where no one compound is exclusive to a single species [20], and in some instances, the differences between species are largely due to the changes in the relative amounts of the compounds rather than differences in the presence or absence of the compound. This could suggest that the chemical differences between close relatives is more subtle in these insects than in *Tetragnatha* spiders. This may not be surprising given that many of these insects use a multitude of other cues to communicate and may rely on the combinatorial effect of multimodal signalling to recognize appropriate mates [9,45–49]. Although many spiders use vibratory communication, either through web-borne or substrate-borne vibrations, sometimes using stridulatory structures [35–37], *Tetragnatha* spiders lack an obvious courtship ritual such as web plucking or stridulation [30,34,50]. Our findings indicate that *Tetragnatha* spiders can make use of chemical signals to recognize conspecifics without the use of vibratory or visual signals.

Chemical profiles between sympatric Hawaiian *Tetragnatha* showed particularly striking differences in the specific identity of methyl ethers found in the silk extracts, with each species having a unique combination of methyl ethers. In comparison, allopatric species had noticeable overlap in their profiles with one instance of complete overlap in the profile of a web-building and spiny leg species from two separate sites. This pattern of reproductive character displacement suggests a role for reinforcement [51,52] with stronger differentiation between taxa that are sympatric versus those that are allopatric [53–55]. In Hawaiian *Tetragnatha* spiders, the greatest structural difference in the methyl ethers were found in the sympatric sibling species of the web-building clade, *T. stelarobusta*, *T. trituberculata* and *T. eurychasma* where they differed significantly in the size of the compound, methyl branch number, ω-branch position, and the chain elongation (electronic supplementary material, S7 and S11). Although *T. trituberculata* had methyl ethers that were structurally intermediate compared to the extremes of *T. stelarobusta* and *T. eurychasma*, the structural characteristics were still significantly diverged from either extreme. Less substantial but still significant, differences were also found between the closely related sympatric species *T. waikamoi* and *T. kamakou* from the spiny leg clade. While the results highlight the potential role of reinforcement, it is unclear how much signal divergence might have preceded the co-occurrence.

Lastly, a key component to understanding the mechanisms of reproductive isolation is how recognition cues change and evolve between populations to understand how these cues may eventually lead to reproductive isolation between these populations. In our study, we detected differences in the chemical profile between two populations of *T. stelarobusta*. The relative amount of methyl ethers differed such that the most abundant compounds were those that differed the most from compounds found in the profile of other closely related sympatric species (figure 4). In chemical signals, the extrinsic environment, often the diet, has been shown to play an important role in driving the divergence of chemical profiles [56–59]. Strong dietary and/or environmental associations that differ may produce divergent chemical signals despite geographical overlap [17,59], or conversely, similar associations may produce more shared compounds across species. Although we cannot rule this out, diet does not seem to play a crucial role in the chemical signals of *Tetragnatha* spiders as the chemicals significantly differed even between species that share dietary preferences (as seen in the spiny leg species and some web-building species) and the same exact chemical signal was found in *T. filiciphilia* and *T. waikamoi*, a web building and spiny leg species that differ in their diet [60]. Moreover, the two sites in our study, Upper Waikamoi (a closed Ohi'a dominated mesic forest) and Lower Waikamoi (a closed Koa and Ohi'a dominated mesic forest) [61] fell within the same overall forest type with roughly similar rainfall, though the higher elevation at the upper site (1900–2000 m) compared to the lower (1400 m) is associated with a lower temperature. Our results seem to align more with previous work showing that recognition cues between populations vary according to the assemblage of close relatives co-occurring at a given site and the associated complexity of the biotic signalling environment [62–67]. Thus, our results are congruent with the hypothesis that chemical profiles may by shaped by reproductive character displacement due to the composition of species within the community.

In conclusion, adaptive radiations have been crucial in helping develop our understanding of the mechanisms involved in speciation. This study highlights the key role of chemistry in species recognition among an adaptive radiation of Hawaiian spiders and highlights the prominent role of chemical signals in adaptive radiation, providing a mechanism for definitive species recognition even in the absence of other possible cues. Future work using the island chronology will allow us to examine the relative timing of signal divergence and associated reproductive isolation, relative to ecological shifts, in fostering speciation [68].

## 4. Methods

### (a) Study organisms

Adult female, adult male and juvenile spiders of 11 different Hawaiian *Tetragnatha* species were collected from The Nature Conservancy of Hawaii's Waikamoi Preserve on the Hawaiian island of Maui during the summers of 2016–2020. The majority of spiders were collected from two sites: Upper Waikamoi at 1800–1950 m and Lower Waikamoi at 1380–1500 m. At Upper Waikamoi, *T. eurychasma*, *T. kamakou*, *T. quasimodo*, *T. stelarobusta*, *T. trituberculata* and *T. waikamoi* can be found in sympatry while at Lower Waikamoi *T. brevignatha*, *T. filiciphilia*, *T. quasimodo*, *T. restricta*, *T. stelarobusta* and *T. waikamoi* can be found in sympatry [27]. On the other hand, *T. acuta* was collected at a higher elevation site just above Upper Waikamoi at around 2000 m, while the undescribed ‘Crater Elongate’, a dry habitat specialist, was collected along the Haleakala Supply Trail and inside Haleakala Crater at 2000–2200 m. California *Tetragnatha* spiders, *T. pallescens* and *T. versicolor*, were collected from Bodega Marine Reserve and Angelo Coast Range Reserve respectively. Once collected, specimens were housed in modified 11 × 7 × 6 cm Tupperware containers with wet cotton balls for moisture and a plastic stick for hiding. The Hawaiian spiders were placed in a large incubator set to a thermal cycle of 10°C to 15°C and a 12 h light and 12 h dark cycle while the California spiders were housed at room temperature with a 12 h light and 12 h dark cycle. All spiders were fed once a week with roughly four *Drosophila hydii* or eight *Drosophila melanogaster* each.

### (b) Silk and silk extract choice trials

To assess whether males of Hawaiian *Tetragnatha* spiders could discriminate conspecific from heterospecific females using silk produced by the females, dragline (major ampullate) silk strands of conspecific and heterospecific females were presented to male spiders in a silk choice trial (figure 1*a*). We used the same protocol as that described in Adams *et al.* [42]. The draglines naturally produced from the females were placed so that the silk strands were attached to the horizontal stick in a Y-formation. A male was then placed on the opposite end of the horizontal stick facing the silk strands. If the male did not move from this position within 30 min, the male was removed and replaced with a new male. The male was considered to have made a choice when he moved off the stick and walked up one of the silk strands. All trials were conducted in the dark under a dim red light to mimic nocturnal conditions and recorded using a smartphone camera. The videos were checked to see if the males touched both silk strands since direct contact with the silk was considered necessary to perceive contact chemical signals. A total of 17 trials was conducted with *T. brevignatha*, 21 trials with *T. eurychasma*, 17 trials with *T. quasimodo*, 20 trials with *T. stelarobusta*, 14 trials with *T. trituberculata* and 16 trials with *T. waikamoi* males. For each species tested, conspecific females from the same site and sympatric heterospecific females from the same site were chosen (specific combinations listed in electronic supplementary material, S1). Heterospecific species from the same clade (spiny leg clade or web-building clade) were chosen for all species except for *T. brevignatha* and *T. stelarobusta*.

To assess whether males of Hawaiian *Tetragnatha* spiders could discriminate conspecific from heterospecific females using chemical signals alone, chemicals from the dragline silk of conspecific and heterospecific females were extracted with dichloromethane (DCM, analytical grade) and the extracts were presented to males on cotton threads in a silk extract choice trial (figure 1*b*). To make the silk extracts, dragline silk that was naturally deposited inside the Tupperware enclosures was collected by wrapping the silk around a clean metal spatula and then pushing it into a tight ball using clean tweezers. The silk ball was then placed in a 2 ml glass vial (Agilent Technologies) and soaked in 0.2 ml of DCM for 30 min. After 30 min, clean tweezers were used to remove the silk ball, the vials were closed and secured with parafilm, and the extracts stored at −20°C until further use. Behavioural trials conducted with these silk extracts were performed according to the protocol described in Adams *et al.* [42]. Two pieces of cotton thread were attached to the bamboo sticks to form a Y-shape. Silk extracts of the females were dispensed with a glass pipette onto each cotton thread. The extracts were then allowed to set for 1 min, giving time for the DCM to evaporate. A male was placed at the opposite end of the bamboo stick in the same manner as in the silk choice trials. The male was considered to have made a choice when he moved off the bamboo stick and walked up one of the cotton threads. All trials were conducted in the dark under red light and recorded using a smartphone camera. A total of 23 trials were conducted with *T. brevignatha*, 21 trials with *T. eurychasma,* 30 trials with *T. quasimodo,* 29 trials with *T. stelarobusta,* 17 trials with *T. trituberculata* and 25 trials with *T. waikamoi* males. As with the silk choice trials, for each species tested in the silk extract choice trials, silk extracts of conspecific females from the same site and silk extracts of sympatric heterospecific females from the same site were chosen (electronic supplementary material, S1). Finally, chi-square analyses were performed to evaluate male choice for both the silk choice trials and silk extract choice trials.

### (c) Chemical analysis of silk extracts

Dragline silk extracts of *Tetragnatha* spiders were analysed by GC-MS (Agilent 7890A/5975C). Dragline extracts from 22 *T. acuta*, 29 *T. brevignatha*, seven *T. eurychasma*, 23 *T. filiciphilia,* 11 *T. kamakou*, seven *T. pallescens*, 33 *T. quasimodo*, eight *T. restricta*, 28 *T. stelarobusta*, 21 *T. trituberculata*, 18 *T. versicolor*, 30 *T. waikamoi* and five ‘Crater Elongate’ were analysed. The gas chromatography was fitted with a fused silica capillary column (Agilent, DB-5MS, 30 m × 0.32 mm × 0.25 µm) with helium as the carrier gas. Extracts were analysed in splitless mode, with a column oven temperature program of 50°C for 5 min, increased by 5°C min^−1^ to 320°C. Injector and transfer line temperatures were maintained at 250°C.

GC-MS data were analysed in OpenChrom [69] by detecting chromatogram peaks using the First Derivative Peak Detector feature at the highest threshold. Peak integration areas and linear retention indices were calculated using the Trapezoid Peak Integrator and Retention Index Calculator features respectively. Previous work showed that methyl ethers are the most abundant compound found in the *Tetragnatha* spider silk extracts and were species-specific [42]. As such, in this study, peaks with a strong *m/z* 45 were identified as methyl ethers [41] and added to a user-built methyl ether library. Methyl ether peaks were aligned manually, based on retention time, retention index and visual comparison with spectra in the library. For each individual chromatogram, relative peak contributions of each methyl ether were calculated by dividing the peak area of a specific methyl ether by the sum of the peak areas of all methyl ethers found in the sample.

### (d) Structural analysis of compounds

To assess the structural evolution of the methyl ethers found across the spiders, the structures of the most common methyl ethers found in each species were determined by the derivatization of the methyl ethers in two separate reactions using previously established procedures in Adams *et al.* [42]: transformation into cyanides via iodination [43] and separate oxidation with RuO_4_ into methyl esters [44]. Using the information from the derivatives, the total carbon number including the methoxy group, the methyl branching pattern, the ω-branching pattern represented as the methyl branching pattern counted from the opposite end of the methoxy group, the carbon backbone length, and the elongation amount represented as the number of carbons between the methoxy group and the methyl branch closest to the methoxy group was determined. ω-Branching patterns were examined in this study since these methyl ethers are most likely to be derived from fatty acid biosynthesis and are proposed to be constructed from the end opposite to the methoxy group, the ω-end, similar to insect hydrocarbons or long-chain alcohols [70,71]. Average values for each structural category were then calculated for every species by averaging the structural properties of the methyl ethers found in all the individual profiles of a given species.

### (e) Statistical analysis of spider chemicals

To assess whether the chemical profiles of Hawaiian *Tetragnatha* exhibit species-specific patterns both in the identity and abundance of methyl ethers, a non-metric multidimensional scaling (NMDS) ordination plot was created using the individual-level dataset in the *vegan* package in R run with two dimensions and 100 iterations [72,73]. Shepard plots were created to see goodness-of-fit. To test for significant differences in chemical profiles between species, permutational multivariate analysis of variance using distance matrices (PERMANOVA) was performed using the adonis function in the *vegan* package [72]. Since PERMANOVA is sensitive to differences in intra-group dispersions [74], an analysis of multivariate homogeneity of group dispersions (PERMDISP) was performed using the betadisper and permutest functions, also in the *vegan* package. All methyl ether profile dissimilarities were calculated using the Bray–Curtis index. Significance was assessed via 999 permutations. Lastly, to assess whether there were differences in the chemical profiles between the sexes and populations within a species, PERMANOVA and PERMDISP analyses were also performed on species grouped by sex (male versus female in *T. acuta, T. brevignatha, T. filiciphilia, T. kamakou, T. quasimodo, T. stelarobusta, T. trituberculata* and *T. waikamoi*) and population location (Upper versus Lower Waikamoi in *T. stelarobusta*). To compare the chemical distance between sympatric and allopatric species pairs, a Kruskal–Wallis and Flingner–Killeen test was performed in R using the Bray–Curtis distance between all species pairs.

To assess whether the chemical profile of closely related species differ more than distantly related species, a reduced phylogenetic tree modified from Kennedy *et al.* [33] was projected onto the two-dimensional chemical space defined by the NMDS plot. The position of the end nodes in chemical space were calculated by taking the centroid of the points plotted on the NMDS for each species, and phylogenetic relationships were depicted through solid black lines extending vertically through time. The end points were coloured according to the phylogenetic branch length to the nearest node corresponding to each species using the ChronoPTS2D function in the ‘*evoldiver*’ package [75] combined with the package ‘*rgl*’ [76] to generate a rotating three-dimensional gif. To visualize the divergence pattern of the chemical profiles through the phylogeny, a disparity through time (DTT) plot was used. The DTT plot visually represents the average relative disparity of each subclade at a given time in the phylogeny by dividing the average disparity of all subclades by the average disparity of the entire clade [77].

A table of the most common methyl ethers were made for each species by listing methyl ethers that were found in more than 50% of the individuals in each species, and those that had an average relative abundance greater than 50%. This ensured that compounds found in large amounts as well as those found in lower amounts but found consistently between individuals of a given species were all represented. For species with significant difference between male and female methyl ether profiles (as determined by ADONIS), a shortlist of methyl ethers for each sex was determined. The two lists were then combined to give a species-level shortlist.

### (f) Statistical analysis of compound structure

To analyse the similarities in the structural patterns of the most common methyl ethers found across the species, maximum common substructure (MCS) was used as a metric for chemical similarity [78,79]. MCS calculations were conducted on the entire structure of the methyl ethers as well as the structures without the methoxy functional group. The MCS and subsequently the Tanimoto coefficient [78] were then computed for all pairwise combinations of the most common methyl ethers. To yield a dissimilarity value between chemical pairs, the Tanimoto coefficient was subtracted from 1. A distance matrix was created using the chemical-pair dissimilarities. All MCS calculations were performed using the R package ‘*fmcsR*’ [79]. Hierarchical clustering based on the distance matrix was used to construct a dendrogram and group the methyl ethers into clusters based on their structural similarity. The optimal number of clusters was determined by silhouette analysis. Cluster stability was assessed via bootstrapping with 1000 resamples using the function clusterboot in the R package ‘*fpc*’ [80]. The ancestral state reconstruction of the odd and even ω-branching patterns found in the species was performed using the R toolkit ‘MBASR’ [81] run for 10 000 generations and sampled every 100 generations. Finally, character states of the total carbon number, number of methyl branches, and elongation through the phylogeny were visualized using a maximum-likelihood approach implemented using the function ‘contMap’ from the ‘*phytools*’ package [82,83], and the traitgram visualized using the ‘phenogram’ function in the ‘*phytools*’ package [82].

## Data Availability

Further information and requests for resources should be directed to and will be fulfilled by the lead contact, S.A.A. (seira.adams@berkeley.edu). All chemical extractions generated in this study were used for GC-MS analysis and therefore none are available for loan or use. All data and code used have been deposited on Dryad (doi:10.6078/D1CB04) [84]. Supplementary material is available online [85].
